# Investigating post-traumatic stress disorder (PTSD) and complex PTSD among people with self-reported depressive symptoms

**DOI:** 10.3389/fpsyt.2022.953001

**Published:** 2022-10-19

**Authors:** Hong Wang Fung, Wai Tong Chien, Stanley Kam Ki Lam, Colin A. Ross

**Affiliations:** ^1^Department of Social Work, Hong Kong Baptist University, Kowloon Tong, Hong Kong SAR, China; ^2^The Nethersole School of Nursing, Faculty of Medicine, The Chinese University of Hong Kong, Shatin, Hong Kong SAR, China; ^3^The Colin A. Ross Institute for Psychological Trauma, Richardson, TX, United States

**Keywords:** depression, post-traumatic stress disorder, complex PTSD, trauma, mental health

## Abstract

**Background:**

Trauma has been increasingly linked to depression. Previous studies have suggested that comorbid post-traumatic stress disorder (PTSD) may be associated with poor outcomes in depression treatment. However, the prevalence and correlates of ICD-11 PTSD and complex PTSD (CPTSD) in people with depression remain unclear.

**Methods:**

This study examined the prevalence and correlates of ICD-11 PTSD and CPTSD in an online convenience sample of 410 adults from 18 different countries/regions who reported clinically significant levels of depressive symptoms (indicated by a Patient Health Questionnaire-9 score ≥10).

**Results:**

According to the International Trauma Questionnaire results, 62.68% of participants met the ICD-11 criteria for PTSD/CPTSD (5.6% PTSD, 57.1% CPTSD). Participants with CPTSD reported more types of trauma and higher levels of interpersonal stress than those without PTSD. Participants with CPTSD also reported higher levels of mental health problems, including depressive, dissociative and psychotic symptoms, than those without PTSD. Only disturbances in self-organization (DSO) symptoms but not classical PTSD symptoms had a significant relationship with depressive symptoms, when other major variables (including trauma, interpersonal stress, and comorbid psychotic and dissociative symptoms) were controlled for.

**Conclusions:**

Trauma-related symptoms should be regularly screened for in clients who report depressive symptoms. Depressed clients who have comorbid trauma disorders have more trauma and interpersonal stress and exhibit more severe mental health problems. They may require specific trauma-focused interventions in addition to standard depression treatments.

## Introduction

### Depression as an important public mental health issue

Depression is one of the most common mental health problems in the world, and it is also a major cause of disability (1). Depression is said to be the largest factor contributing to global disability as measured by years lost to disability (YLD) (2). Although depression has been increasingly recognized as a significant public health issue, our understanding of this mental health condition and its treatment needs is still limited. For example, the very influential, widely cited serotonin hypothesis of depression has been found to have very limited to no empirical evidence in a recent systematic umbrella review (3). This implies that the mechanisms behind the potential effects of antidepressants remain unclear. In addition, despite the potential advantages of using psychotherapy to treat depression, as Markowitz et al. (4) said, “treatment selection research is nascent and complex” and little is known about how to match patient characteristics with specific interventions. Although cognitive behavioral therapy (CBT) is generally believed to be effective for depression, a systematic review indicated that we still have limited understanding about which components work for whom (5). Walsh et al. (6) reviewed 75 randomized controlled trials and reported that about 50% of patients with depression did not respond to antidepressants. As revealed in a review of recent meta-analyses, the effects of current psychotherapies (standardized mean difference [SMD] = 0.11 to 0.61) and pharmacotherapies (SMD = 0.19 to 0.41) for depressive disorders in comparison to placebo or treatment-as-usual are generally small (7).

### People with depression as a heterogeneous population

To improve our understanding regarding what works for whom, it is important to take individual needs into account. One major reason for the above-mentioned unfavorable treatment outcomes is that people with depression are a highly heterogeneous group of service users (8, 9). Current diagnostic and treatment approaches focus on the symptoms of depression rather than its underlying causes or triggers. However, clinically, one patient may develop depression after traumatic events, while another patient may have infection or inflammation-induced depression—although the depressive symptoms may be phenomenologically similar, their intervention needs may be entirely different (10). In other words, the same set of depressive symptoms may have different causes, and thus require different interventions (11). Therefore, to improve treatment outcomes for depression, it is important to understand the individual causes of the symptoms and identify which subtype the depression may belong to Rantala et al. (10) and Maj et al. (12).

### A possible post-traumatic subtype of depression

Among different possible subtypes of depression, a post-traumatic subtype characterized by the presence of co-occurring post-traumatic reactions has been receiving increasing support in the literature. Trauma, post-traumatic stress disorder (PTSD) and depression have a complex relationship. A meta-analysis indicated that childhood traumatic events, especially emotional maltreatment, are associated with adulthood depression (13). Trauma may lead to depressive symptoms (14). Disaster-affected young people also have a higher prevalence rate of depression than controls (15). From an evolutionary and neuroscience-based perspective, Rantala et al. (10) proposed a number of possible subtypes of depression (e.g., infection-induced depression, postpartum depression, seasonally-related depression) and some major subtypes could be induced by trauma and stress. In order to survive, some organisms may avoid situations in which the danger is likely to occur again, resulting in symptoms of depression and social withdrawal. Moreover, stress may lead to inflammation, which is also associated with depression (16). It is important to note that trauma-related depression may require interventions (e.g., trauma-focused psychotherapy) that are different from other subtypes of depression (e.g., seasonally-related depression may require light therapy) (10). Therefore, the comorbidity of depression and trauma-related disorders has been an important topic of research in recent years; however, most comorbidity depression-PTSD research focused on persons with PTSD who also have a subtype with depression (17). In terms of people with depression as the primary concern, one study found that comorbid PTSD was associated with lower odds of remission and higher odds of persistent depressive symptoms at 6 months after collaborative care management for depression (18). A comorbid PTSD is also listed as a negative prognostic factor in depression by other authors (19). More importantly, when depression co-occurs with PTSD, trauma-focused treatments should be considered because trauma-focused treatments can effectively treat depressive symptoms while depression-focused treatments may not reduce PTSD symptoms (20). This literature indicates that a considerable subgroup of people suffering from depression may have co-occurring or underlying trauma-related difficulties. Although many studies investigated depression in people with PTSD, very little is known about PTSD symptoms in people with depression. Therefore, it is important to understand the prevalence and correlates of trauma-related problems in people who present with depressive symptoms.

### Existing knowledge gaps

Despite the clinical significance of a comorbid PTSD in people with depression, there are some major limitations and knowledge gaps in the current literature. First, as mentioned, while there are many studies of the prevalence of comorbid major depression in people with PTSD (which is said to be 52%, although it also varies across populations, see the meta-analysis of (21), very few studies have reported the prevalence of comorbid PTSD in people with depression. As reported in a small number of studies, the rate of PTSD could be as high as 36 to 61.0% among people with depression (22–24). Second, to our knowledge, no study has investigated complex PTSD (CPTSD) among people with depression. CPTSD is a newly recognized trauma-related mental disorder in ICD-11, and it covers disturbances in self-organization (DSO) symptoms (i.e., affective dysregulation, negative self-concept, and disturbances in relationships) in addition to classical PTSD symptoms (i.e., re-experiencing, avoidance, and sense of current threat) (25). Karatzias et al. (26) reported that this new ICD-11 diagnosis was more common than ICD-11 PTSD in a population study (12.9% vs. 5.3%), and that CPTSD was more strongly associated with an increasing risk of having major depression (89.0% vs. 48.2%) and anxiety (86.0% vs. 30.4%) than was PTSD.

### The present study

Given these two knowledge gaps, the primary goal of the present study was to examine the prevalence of PTSD and CPTSD in a sample of people with clinically significant levels of depressive symptoms. In line with similar studies on CPTSD in other populations [e.g., (27, 28)], we also compared those with and without PTSD/CPTSD in our sample on major clinical variables that we assessed, including trauma histories, interpersonal stress, depressive symptoms, dissociative symptoms, and psychotic symptoms.

In addition, some studies found that PTSD symptoms could predict depressive symptoms (29, 30), but these studies did not take ICD-11 CPTSD symptoms into account. Less is known about whether other types of post-traumatic symptoms (i.e., DSO symptoms) are also associated with depressive symptoms. Therefore, this study also examined the association of classical PTSD symptoms and DSO symptoms with depressive symptoms.

## Methods

### Participants and procedures

This study analyzed data from a cross-sectional survey study which investigated life experiences and mental health problems in a convenience sample of people who reported clinically significant depressive symptoms. From November 2021 to February 2022, we recruited potential participants through social media platforms related to mental health in general or depression in particular. Social media advertising was used to facilitate the recruitment. A recruitment poster was provided to invite potential participants to complete a password-protected Google Form.

Participants were those who met the following enrollment criteria: (1) aged 18 or above; (2) provided informed consent to participate; (3) were able to access the Internet and read English; and (4) who endorsed the question, “have you ever suffered from any depressive emotions?” Participants who endorsed the following questions were excluded from the online survey: “Do you have immediate need for professional help, or do you have a currently unstable mental health status?” “Do you have a diagnosed reading disorder, dementia or intellectual disabilities?” “Have you had recurrent suicidal ideation, suicidal attempts or homicidal plans in the past 2 weeks?” These individuals were advised to seek professional help and not to complete the online survey. Furthermore, only participants who reported moderate-severe levels of depressive symptoms (score of 10 or above) on the Patient Health Questionnaire-9 [PHQ-9 (31)] were included in the study. The methodology and part of the data have been reported elsewhere (32).

Regarding the research questions and hypotheses, as noted, this study aimed to investigate the prevalence of PTSD/CPTSD in this sample. In addition, we also examined the relationship between PTSD/CPTSD symptoms and interpersonal stress, dissociation and psychotic symptoms because these experiences are commonly associated with trauma (33–35).

### Ethical considerations

The study received ethical approval from the Survey and Behavioural Research Ethics Committee at The Chinese University of Hong Kong. The online survey was anonymous and voluntary. Before participants started the survey, they were asked to provide online informed consent to participate in the survey, after reading the detailed information sheet and consent form. A list of hotline mental health and support services was provided before and after the survey. Participants were advised to seek professional help and not to complete the survey if they needed any immediate help or had unstable mental health problems.

### Measures

In the web-based survey, participants completed questions about clinical and sociodemographic characteristics (e.g., age, gender, education level, employment status, prior psychiatric diagnoses, and use of mental health services) and a set of self-report measures as follows:

#### Patient health questionnaire-9 (PHQ-9)

The PHQ-9 is a 9-item self-report measure of depression; it can be used to make a provisional diagnosis of major depressive disorder (36). The PHQ-9 has good internal consistency (α = 0.86), test-retest reliability (r = 0.84), and concurrent validity (r = 0.77) with the Beck Depression Inventory (BDI-II) (37, 38). Manea et al. (31) conducted a meta-analysis of the PHQ-9 and reported that 8 to 11 were acceptable PHQ-9 cutoff scores for detecting major depressive disorder.

#### Brief betrayal trauma survey (BBTS)

The BBTS is a 24-item self-report measure that assesses both childhood and adulthood traumatic events (39). It assesses 12 different types of trauma before and after age 18. Betrayal trauma is perpetrated by someone with whom the victim is close or is dependent upon (e.g., family members), while non-betrayal trauma is not (40). The BBTS was reported to have good test-retest reliability over 3 years in a community sample, and the agreement rate between the two test administrations for childhood and adulthood trauma was 83 and 75%, respectively (39).

#### International trauma questionnaire (ITQ)

The ITQ is an 18-item self-report measure of ICD-11 PTSD and CPTSD (41). It assesses both classical PTSD symptoms and DSO symptoms. In particular, six of the 18 items on the ITQ comprise the DSO scale, which consists of three subscales with two items each: affective dysregulation (AD); negative self-concept (NSC); and disturbances in relationships (DR). The NSC items are: “I feel like a failure” and “I feel worthless”; the AD items are, “When I am upset, it takes me a long time to calm down” and “I feel numb or emotionally shut down”; and the DSO items are, “I feel distant or cut off from people” and “I find it hard to stay emotionally close to people”. The ITQ has good to excellent internal consistency (α = 0.89 to.94) and excellent concurrent validity with the PTSD Checklist for DSM-5 (r = 0.89) (42). The ITQ can be used to diagnose PTSD and CPTSD according to ICD-11 rules (41), when an individual has encountered any traumatic events (in this study, participants should have endorsed at least one traumatic event on the BBTS in order to meet the ICD-11 PTSD/CPTSD criteria).

#### Bergen social relationships scale (BSRS)

The BSRS is a 6-item self-report measure of chronic interpersonal stress. The BSRS has satisfactory internal consistency (α = 0.73), and its factor structure was found to be invariant across different cultures tested (43). Previous studies demonstrated that the BSRS was positively associated with psychological distress and that the effects of the stress were independent of social support (44).

#### Multidimensional scale of perceived social support-family support (MSPSS-FS)

The MSPSS is a 12-item self-report measure of perceived social support. It was reported to have good internal consistency (α = 0.79) and good construct validity with the Social Support Behaviors Scale (r = 0.13 to.77) (45, 46). Its factor structure was further confirmed in Canty-Mitchell and Zimet (47). The Family Support subscale has 4 items and can specifically assess the level of perceived family support.

#### Multiscale dissociation inventory (MDI)

The MDI is a 30-item self-report measure of psychoform dissociative symptoms (48). This measure can assess six different types of psychoform dissociative symptoms, which are moderately intercorrelated (mean r = 0.39) and internally consistent (α = 0.74 to 96) (49). Most factors of the MDI had good convergent validity with other dissociation measures (β = 0.22 to.49), except for the emotional constriction subscale (48).

#### The 5-item somatoform dissociation questionnaire (SDQ-5)

The SDQ-5, which is a brief version of the original 20-item SDQ, is a self-report measure of somatoform dissociative symptoms (sometimes referred to as medically unexplained physical symptoms) (50, 51). The SDQ-5 has good internal consistency (α = 0.80) and excellent diagnostic validity (a cutoff score of ≥ 8 could detect patients with a dissociative disorder with a sensitivity of 94% and a specificity of 96%) (51).

#### The positive and negative symptoms frequency subscales of the community assessment of psychic experiences (CAPE-P and CAPE-N)

The CAPE is a 42-item self-report measure of psychotic symptoms (52). The CAPE has three subscales that can be used to assess psychotic symptoms (20 items) and negative symptoms (14 items) of psychosis and depressive symptoms. A meta-analysis indicated that both the CAPE-P (mean α = 0.84) and the CAPE-N (mean α = 0.81) have good internal consistency, and that the three-factor structure of the CAPE was supported (53).

### Data analysis

SPSS 22.0 was used to analyze the data. PTSD and CPTSD were assessed and diagnosed according to ICD-11 rules using the ITQ. Trauma, interpersonal stress and perceived family support were assessed using the BBTS, BSRS and MSPSS-FS, respectively. Depressive symptoms were assessed using the PHQ-9. Psychoform and somatoform dissociative symptoms were assessed using the MDI and SDQ-5, respectively. Positive and negative psychotic symptoms were assessed using the two CAPE subscales.

Descriptive statistics were used to summarize the clinical and sociodemographic characteristics and the frequency of PTSD/CPTSD in the participants. We then examined the differences between the participants with and without PTSD/CPTSD on their trauma, interpersonal stress, family support, depressive symptoms, dissociative symptoms and psychotic symptoms using one-way ANOVA. *Post-hoc* testing was conducted using a Bonferroni correction. Moreover, a Pearson's correlation test was used to examine the relationships of PTSD, DSO, and depressive symptoms with other included variables. To examine the association of both classical PTSD symptoms and DSO symptoms with depressive symptoms, we conducted a hierarchical multiple regression analysis to predict depressive symptoms from PTSD and DSO symptoms, after controlling for variables which had a significant correlation with depressive symptoms (i.e., childhood betrayal trauma, childhood non-betrayal trauma, adulthood betrayal trauma, interpersonal stress, psychoform and somatoform dissociative symptoms, and positive and negative symptoms of psychosis. In order to examine whether DSO symptoms would be associated with depressive symptoms above and beyond the effects of classical PTSD symptoms, these symptoms were put in the model one by one.

## Results

### Sample characteristics and prevalence of PTSD and CPTSD

A total of 468 participants provided valid responses and 410 of them met all inclusion criteria (including scored 10 or above on the PHQ-9). Most of them were female (91.7%). Their ages ranged from 18 to 65 (M = 25.3; SD = 8.2). This is a regionally diverse sample as they reported living in 18 different countries/regions, including: 24.1% from the United Kingdom, 23.4% from Canada, 17.3% from Singapore, 12.9% from the United States, and <7% from each of the remaining 14 countries/regions.

About one-fourth of the participants (23.9%) were currently seeing a psychiatrist. Self-reported psychiatric diagnoses included: major depressive disorder (41.2%), other depressive disorders (37.3%), anxiety disorders (63.4%), PTSD (16.8%), and CPTSD (13.7%). Table 1 reports the sample characteristics.

**Table 1:** Sample characteristics (N = 410).

**Variables**	**Mean**	**SD**	**Percentage**
Age	25.26	8.21	
Gender			91.7%
Marital status (married)			17.8%
Education level (Bachelor's degree or above)			39.8%
Being unemployed			41.7%
Financially dependent on partners, friends, family or other people			52.2%
Financially dependent on social welfare			16.1%
Prior major depression diagnosis			41.2%
Currently seeing a psychiatrist			23.9%
Prior PTSD diagnosis			16.8%
Prior CPTSD diagnosis			13.7%
Any childhood trauma			88.78%
Any adulthood trauma			87.56%
Interpersonal stress (possible range: 6 to 24)	15.86	3.79	
Perceived family support (possible range: 0 to 7)	3.57	1.62	
Depressive symptoms (possible range: 3 to 27)	19.37	4.53	
PTSD symptoms (possible range: 0 to 24)	14.99	5.49	
DSO symptoms (possible range: 0 to 24)	18.31	4.06	
Psychoform dissociative symptoms (possible range: 30 to 150)	77.27	25.36	
Somatoform dissociative symptoms (possible range: 5 to 25)	6.76	2.38	
Positive symptoms of psychosis (possible range: 20 to 80)	35.50	10.80	
Negative symptoms of psychosis (possible range: 14 to 56)	37.70	8.30	

On the BBTS, participants reported an average of 3.15 (SD = 2.30) types of childhood trauma and 2.78 (SD = 2.25) types of adulthood trauma; 95.1% reported at least one lifetime traumatic event.

According to the ITQ results, 5.6% of participants met the ICD-11 criteria for PTSD, and 57.1% met the criteria for CPTSD. In total, 257 participants (62.68%) had either PTSD or CPTSD.

### Group differences

One-way ANOVA revealed that there were a number of clinical differences among three subgroups (see Table 2). *Post-hoc* testing showed that participants with CPTSD reported more types of childhood and adulthood trauma (*p* = < 0.001) and higher levels of interpersonal stress (*p* = 0.001) than those without PTSD. Participants with CPTSD also reported more depressive symptoms (*p* < 0.001), psychoform and somatoform dissociative symptoms (*p* < 0.001), and positive (*p* < 0.001) and negative (*p* = 0.001) psychotic symptoms than those without PTSD. Nevertheless, the three subgroups did not significantly differ in the levels of perceived family support (*p* > 0.250).

**Table 2:** Differences between participants with and without (complex) post-traumatic stress disorder (C) PTSD (*N* = 410).

	**A: Participants without** **PTSD (*****n*** = **153)**		**B: Participants with** **PTSD** **(*****n*** = **23)**		**C: Participants with** **CPTSD (*****n*** = **234)**		**F**	**Post-hoc**	**p**
**Variables**	**M**	**SD**	**M**	**SD**	**M**	**SD**			
Age	24.99	9.09	26.26	7.39	25.34	7.68	0.261	A = B = C	0.770
Childhood betrayal trauma	1.22	1.27	1.57	1.31	1.97	1.35	15.208	A < C	0.000
Childhood non-betrayal trauma	0.59	0.85	1.00	1.13	1.19	1.15	14.982	A < C	0.000
Adulthood betrayal trauma	0.95	1.09	1.22	1.00	1.64	1.23	16.414	A < C	0.000
Adulthood non-betrayal trauma	0.59	0.82	1.17	1.11	1.12	1.20	11.999	A < B, A < C	0.000
Interpersonal stress	15.02	3.54	15.22	3.94	16.47	3.83	7.305	A < C	0.001
Perceived family support	3.64	1.48	4.09	1.74	3.47	1.69	1.750	A = B = C	0.175
Depressive symptoms	17.84	4.55	16.57	3.24	20.64	4.19	24.965	A < C, B < C	0.000
Psychoform dissociative symptoms	67.58	21.19	64.52	22.37	84.87	25.55	27.811	A < C, B < C	0.000
Somatoform dissociative symptoms	6.22	1.80	5.96	2.20	7.18	2.64	9.250	A < C	0.000
Positive psychotic symptoms	31.15	7.49	32.30	8.95	38.66	11.70	26.374	A < C, B < C	0.000
Negative psychotic symptoms	36.27	7.65	31.13	8.80	39.28	8.21	14.611	A>B, A < C, B < C	0.000
**Variables**		**Percentage**		**Percentage**		**Percentage**	χ**2**	**df**	**p**
Gender (female)		89.5%		91.3%		93.2%	3.489	4	0.480
Marital status (married)		13.7%		34.8%		18.8%	6.429	2	0.040
Education level (Bachelor's degree or above)		37.9%		47.8%		40.2%	0.860	2	0.650
Being unemployed		43.8%		34.8%		41.0%	2.789	4	0.594
Financially dependent on partners, friends, family or other people		57.5%		39.1%		50.0%	3.761	2	0.152
Financially dependent on social welfare		13.1%		13.0%		18.4%	2.095	2	0.351
Prior major depression diagnosis		36.6%		26.1%		45.7%	5.482	2	0.064
Currently seeing a psychiatrist		17.6%		17.4%		28.6%	7.892	4	0.096
Prior PTSD diagnosis		4.6%		30.4%		23.5%	26.905	2	0.000
Prior CPTSD diagnosis		2.0%		13.0%		1.4%	29.552	2	0.000

We did not observe many significant differences between participants with and without PTSD. Participants with PTSD reported more types of adulthood non-betrayal trauma (*p* = 0.043) and but fewer negative psychotic symptoms (*p* = 0.013) than those without PTSD.

### Relationships between PTSD, DSO, and depressive symptoms

In Table 3, we report the Pearson's correlations of PTSD, DSO and depressive symptoms with other major variables. PTSD, DSO and depressive symptoms had significant positive correlations with all included variables, except for age and adulthood non-betrayal trauma. Only PTSD symptoms were positively correlated with the number of types of adulthood non-betrayal trauma (r = 0.291, *p* < 0.001). Additional information about the partial correlations among the symptom variables is reported in the Supplementary Table.

**Table 3:** Correlations between post-traumatic stress disorder (PTSD), disturbances in self-organization (DSO) symptoms, and depressive symptoms and other clinical variables (*N* = 410).

**Variables**	**PTSD** **symptoms**	**Disturbances in** **self-organization** **(DSO) symptoms**	**Depressive** **symptoms**
Age	0.072	−0.033	−0.017
Childhood betrayal trauma	0.386^***^	0.216^***^	0.132^**^
Childhood non-betrayal trauma	0.344^***^	0.160^**^	0.166^**^
Adulthood betrayal trauma	0.359^***^	0.173^***^	0.112^*^
Adulthood non-betrayal trauma	0.291^***^	0.071	0.063
Interpersonal stress	0.253^***^	0.195^***^	0.212^***^
Depressive symptoms	0.293^***^	0.588^***^	/
Psychoform dissociative symptoms	0.461^***^	0.475^***^	0.471^***^
Somatoform dissociative symptoms	0.286^***^	0.248^***^	0.203^***^
Positive psychotic symptoms	0.370^***^	0.286^***^	0.270^***^
Negative psychotic symptoms	0.238^***^	0.477^***^	0.458^***^

* p < 0.05,

** p < 0.01, and

*** p < 0.001.

We further examined the relationship of PTSD and DSO symptoms with depressive symptoms using a hierarchical multiple regression analysis (see Table 4). After controlling for the variables which had a significant correlation with depressive symptoms, PTSD symptoms were still significantly associated with depressive symptoms (β = 0.106, *p* = 0.036), and the addition of PTSD symptoms to the prediction model (Model 2) led to a statistically significant increase in R^2^ of 0.008, *F*_(1,400)_ = 20.157, *p* < 0.001. In Model 3, we further put DSO symptoms into the model, and we found that DSO symptoms had the strongest relationship with depressive symptoms (β = 0.410, *p* < 0.001). The addition of DSO symptoms to the prediction of depressive symptoms (Model 3) led to a statistically significant increase in R^2^ of 0.114, *F*_(12,400)_ = 32.099, *p* < 0.001. However, in this model, PTSD symptoms (β = 0.038, *p* = 0.420) no longer had a significant relationship with depressive symptoms. Instead, psychoform dissociative symptoms (β = 0.235, *p* < 0.001) and negative psychotic symptoms (β = 0.179, *p* < 0.001) still had a significant relationship with depressive symptoms. In other words, even after controlling for PTSD symptoms and other major variables, DSO symptoms were still the strongest predictor of depressive symptoms in our sample (see Table 4).

**Table 4:** Hierarchical multiple regression predicting depressive symptoms from post-traumatic symptoms (N = 410).

	**Depressive symptoms**											
	**Model 1**				**Model 2**				**Model 3**			
**Variables**	**Partial** **correlation**	**Part** **correlation**	**β**	**p**	**Partial** **correlation**	**Part** **correlation**	**β**	**p**	**Partial** **correlation**	**Part** **correlation**	**β**	**p**
Constant				0.000				0.000				0.000
Childhood betrayal trauma	−0.015	−0.013	−0.016	0.763	−0.030	−0.025	−0.032	0.553	−0.055	−0.042	−0.054	0.275
Childhood non-betrayal trauma	0.035	0.029	0.034	0.485	0.024	0.020	0.023	0.631	0.043	0.032	0.038	0.395
Adulthood betrayal trauma	0.013	0.011	0.013	0.793	−0.003	−0.003	−0.003	0.948	−0.010	−0.008	−0.010	0.836
Interpersonal stress	0.106	0.089	0.095	0.033	0.098	0.081	0.087	0.050	0.086	0.066	0.070	0.085
Psychoform dissociative symptoms	0.299	0.261	0.390	0.000	0.268	0.231	0.356	0.000	0.192	0.148	0.235	0.000
Somatoform dissociative symptoms	−0.087	−0.073	−0.090	0.082	−0.086	−0.071	−0.088	0.086	−0.084	−0.064	−0.080	0.092
Positive psychotic symptoms	−0.096	−0.080	−0.108	0.055	−0.104	−0.086	−0.117	0.038	−0.065	−0.049	−0.067	0.196
Negative psychotic symptoms	0.296	0.259	0.313	0.000	0.301	0.262	0.316	0.000	0.182	0.140	0.179	0.000
Post-traumatic stress disorder (PTSD) symptoms					0.104	0.087	0.106	0.037	0.040	0.031	0.038	0.420
Disturbances in self-organization (DSO) symptoms									0.403	0.334	0.410	0.000
R^2^	0.305				0.312				0.424			
Adjusted R^2^	0.291				0.297				0.409			
F	21.947^**^				20.157^**^				29.329^**^			
ΔR^2^	0.305				0.008				0.112			
ΔF	21.947^**^				4.362^*^				77.287^**^			

* p < 0.05 and

** p < 0.001.

## Discussion

The major findings of this study include the following: first, ICD-11 PTSD and CPTSD were common (62.68% in total) in a sample of people who reported clinically significant levels of depressive symptoms. Second, similar to previous studies on CPTSD in other populations [e.g., (28)], we found that participants with comorbid CPTSD reported more types of trauma, more interpersonal stress, and more comorbid mental health problems than those without comorbid CPTSD, including higher levels of depressive symptoms, dissociative symptoms and psychotic symptoms. Third, while both PTSD and DSO symptoms were positively correlated with depressive symptoms, only DSO symptoms had a significant relationship with depressive symptoms when other major variables (including trauma, stress and comorbid symptoms) were taken into account. This study is among the few studies that have investigated PTSD among people with depression. This is also the first study that examined the prevalence and correlates of ICD-11 CPTSD in this population. Our findings extend previous knowledge about PTSD in depression, and their implications should be further discussed.

First of all, it is important to note that the prevalence of trauma disorders in general, and CPTSD in particular, is high in our sample of people with self-reported depressive symptoms. Although CPTSD is a severe form of PTSD, we found that CPTSD is more common that PTSD. This is, however, not surprising as some recent studies using the ITQ have also reported that the prevalence rate of participants meeting ICD-11 CPTSD criteria was much higher than that of participants meeting ICD-11 PTSD criteria—for example, 54.2% vs. 13.6% in a sample of treatment-seeking veterans who had been diagnosed with a mental health difficulty (*N* = 177) (28), and 30.3% vs. 15.3% in a sample of Danish veterans referred for psychological treatment (*N* = 294) (54). This finding implies that a considerable subgroup of people with depression may be suffering from a condition that requires trauma-focused interventions and that their depressive symptoms may be more or at least in part induced by trauma or its consequences. In our sample, even among participants who met the ICD-11 CPTSD criteria on the ITQ, only 44.9% had a prior PTSD/CPTSD diagnosis. Although this finding is preliminary due to the use of self-report measures, our observation is consistent with previous studies showing that PTSD is sometimes undiagnosed in clinical settings (55, 56). We recommend that mental health service providers screen clients routinely for post-traumatic reactions. When a client presents with both depression and PTSD/CPTSD, trauma-focused treatments should be considered because trauma-focused treatments are first-line treatments for PTSD and because some research shows that depression can be treated using trauma-focused treatments (20, 57). Our findings call for further investigation to examine whether undiagnosed trauma disorders are common among carefully diagnosed patients with depression.

Previous studies showed that people with CPTSD had more mental health comorbidities than those without PTSD [e.g., (28)]. We also found similar results—depressive participants with CPTSD exhibited more severe mental health problems than those without PTSD, including higher levels of depressive and psychotic symptoms. Having said that, the differences between CPTSD and PTSD were not significant in our sample, which is possibly due to the small number of participants in the PTSD group. Previous studies have also shown that psychotic symptoms are not rare among people with trauma-related psychopathology (58, 59). Therefore, when a client presents with severe depression or psychotic symptoms, trauma disorders should be considered as possible comorbid conditions. Although it is beyond the scope of this paper, further research and discussion are needed regarding the order of treatments for people with comorbid depression and trauma disorders. In fact, considering the potentially high prevalence of trauma disorders among people with depression, and since 95.1% of participants reported trauma exposure in our sample, it may be important to provide trauma-informed services (not necessarily trauma-focused exposure-based psychotherapy) so as to acknowledge the impacts of trauma, recognize the trauma responses, and prevent re-traumatization.

In a previous study, Vang et al. (60) analyzed data in two clinical samples and reported that, compared with classical PTSD symptoms, DSO symptoms had a stronger relationship with depressive symptoms. Similarly, we found that DSO but not classical PTSD symptoms were associated with depressive symptoms. It is reasonable to argue that DSO may have predicted depressive symptoms because persons with DSO have higher levels of PTSD symptoms too (61). However, in our regression model, we showed that DSO symptoms were the strongest correlate of depressive symptoms even after controlling for classical PTSD symptoms and other major variables (see Table 4). Having said that, the clinical entity of CPTSD comprises both PTSD and DSO symptoms, and DSO symptoms are also common in people with other disorders, such as borderline personality disorder (62). Therefore, this finding should be interpreted with caution. Besides, negative symptoms of psychosis and psychoform dissociative symptoms were also associated with depressive symptoms. Among different social and mental health variables, DSO symptoms had the strongest relationship with depressive symptoms. One of the possible reasons is that “negative self-concept” (NSC) is included as one DSO symptom in ICD-11 while it is also a feature of depression. The NSC items in the DSO subscale could be thought of as a PTSD-related negative cognition. Within CPTSD, the NSC items are conceptualized as elements of the CPTSD rather than as a separate depressive comorbidity. The NSC items are expected to create a correlation between overall scores on the ITQ and measures of depression like the Beck Depression Inventory (BDI), just as the negative cognition items in the DSM-5 criteria for PTSD would correlate with BDI scores. Nevertheless, the reasons behind the close relationship between DSO symptoms and depressive symptoms require further research and discussion in the future. Based on current findings, it appears that timely identification and management of DSO symptoms and psychoform dissociative symptoms, if any, may be important when working with clients with depression, although the bidirectional relationship between DSO/dissociative symptoms and depression also requires future longitudinal research. It has been demonstrated that trauma therapies are effective in treating CPTSD, including DSO symptoms (63).

Despite the significant implications of the findings, this study has some major limitations. First, the exclusion of participants with a recurrent suicidal risk, immediate need for professional help and/or unstable mental health condition could limit the generalizability of our findings. The use of online methods to recruit a convenience sample of participants also implies that we did not include those who were not willing to participate, and those who could not access the online survey (e.g., inpatients). Second, similarly, given the over-representation of female participants in this study, which was likely due to the use of social media platforms for recruitment (64), our findings may not fully apply to males with depression. In addition, similar findings were obtained when the male participants were removed from the analysis. Thus, further studies using a representative sample of carefully diagnosed patients with depression are needed. Third, given the cross-sectional design, we could not demonstrate causal relationships among the variables tested. Fourth, the sample size of the PTSD subgroup was relatively small. Finally, the use of self-report measures is also a major limitation. Although the ITQ is widely used to assess PTSD/CPTSD in the literature, future studies should use diagnostic interviews to increase the level of evidence regarding the prevalence of trauma disorders in people with depression. However, we used well-validated mental health measures to ensure the validity of the findings. This was also the first study to examine CPTSD in people with depression.

## Conclusion

This study contributes to the literature by reporting the prevalence of PTSD/CPTSD in a sample of people with self-reported depressive symptoms. A considerable subsample met the ICD-11 criteria for CPTSD and they reported more trauma and interpersonal stress and exhibited more comorbid mental health problems than those who had depression but no PTSD. Trauma-related mental disorders should be regularly screened for in mental health service users who report depressive symptoms. Depressed clients who have comorbid CPTSD may require specific trauma-focused interventions in addition to standard depression treatments.

## Data availability statement

The datasets presented in this article are not readily available because due to the nature of this research, participants of this study did not agree for their data to be shared publicly, so supporting data is not available.

## Ethics statement

This study involved human participants and was reviewed and approved by the Survey and Behavioural Research Ethics Committee at The Chinese University of Hong Kong. The participants provided their written informed consent to participate in this study.

## Author contributions

HF, WC, SL, and CR contributed to the conception and design of the study and reviewed and modified the manuscript. HF performed the statistical analysis and wrote the first draft of the manuscript. All authors contributed to manuscript revision, read, and approved the submitted version.

## Conflict of interest

The authors declare that the research was conducted in the absence of any commercial or financial relationships that could be construed as a potential conflict of interest.

## Publisher's note

All claims expressed in this article are solely those of the authors and do not necessarily represent those of their affiliated organizations, or those of the publisher, the editors and the reviewers. Any product that may be evaluated in this article, or claim that may be made by its manufacturer, is not guaranteed or endorsed by the publisher.
