# Supplementary material for: Investigating post-traumatic stress disorder (PTSD) and complex PTSD among people with self-reported depressive symptoms
Source: Front Psychiatry. 2022 Oct 19;13:953001. doi: 10.3389/fpsyt.2022.953001 (PMC9627202; doi:10.3389/fpsyt.2022.953001)
Supplement: Supplementary file 1 [file Table_1.pdf]

Supplementary file

Table S. Partial correlation among mental health symptoms (N = 410)

Variables		1	2	3	4	5	6
1.	Depressive symptoms	1.000					
2.	PTSD symptoms	.235	1.000				
3.	DSO symptoms	.568	.284	1.000			
4.	Psychoform dissociation	.438	.358	.430	1.000		
5.	Somatoform dissociation	.158 [a]	.177	.195	.516	1.000	
6.	Positive symptoms of psychosis	.221	.244	.224	.573	.416	1.000
7.	Negative symptoms of psychosis	.438	.165 [a]	.452	.498	.270	.439

Notes: all $p < .001$, except for [a] $p = .001$

Control Variables: The numbers of childhood and adulthood trauma.
